# Genome Sequencing of Omicron Variants of SARS-CoV-2 Circulating in Bangladesh during the Third Wave of the COVID-19 Pandemic

**DOI:** 10.1128/mra.00381-22

**Published:** 2022-05-31

**Authors:** Munira Jahan, M. Abdullah Omar Nasif, Raad Rahmat, SM Rashed Ul Islam, Saif Ullah Munshi, M. Sharfuddin Ahmed

**Affiliations:** a Department of Virology, Bangabandhu Sheikh Mujib Medical University, Shahbag, Dhaka, Bangladesh; b Bangabandhu Sheikh Mujib Medical University, Shahbag, Dhaka, Bangladesh; c Biotechnology Program, Department of Mathematics and Natural Sciences, School of Data and Sciences, BRAC University, Mohakhali, Dhaka, Bangladesh; DOE Joint Genome Institute

## Abstract

The coding-complete genome sequence of severe acute respiratory syndrome coronavirus 2 (SARS-CoV-2) was obtained from 39 nasopharyngeal swab samples collected in January 2022 from Dhaka, Bangladesh, during the 3rd wave of the COVID-19 pandemic, using Illumina MiniSeq sequencing technology. Sequence analysis showed that all of them belonged to the WHO-designated variant of concern (VOC) Omicron. The presence of different sublineages of Omicron was noted, among which sublineage BA.2 (Nextstrain clade 21L) was the most prevalent one.

## ANNOUNCEMENT

Starting from Wuhan, Hubei Province, China, in late December 2019, the COVID-19 pandemic has devasted the whole world with huge numbers of cases and deaths (1, 2). The causative organism severe acute respiratory syndrome coronavirus 2 (SARS-CoV-2) is a member of the *Betacoronavirus* genus in the *Coronaviridae* family that also contains other human pathogens, SARS-CoV and MERS-CoV (3, 4). In Bangladesh, after a relatively long period of a low number of cases, COVID-19 cases surged rapidly during January 2022 (5). There was a strong demand to know which variants were circulating in the country for public health decision making. In this regard, this study was undertaken.

Nasopharyngeal swab samples were collected from the patients attending the fever clinic of Bangabandhu Sheikh Mujib Medical University, Dhaka, Bangladesh, during January 2022. Real-time reverse transcription-PCR was performed to detect SARS-CoV-2 RNA using the Sansure novel coronavirus (2019-nCoV) nucleic acid diagnostic kit. A total of 39 samples were selected for sequencing (cycle threshold [*C_T_*] of <25 for both ORF1ab and N gene). In short, viral nucleic acid was extracted using the QIAamp viral RNA minikit (Qiagen, Germany). First-strand cDNA synthesis was done using the GoScript reverse transcriptase system (Promega Corporation, USA). Enrichment was done using COVIDSeq primer pool 1 and 2 (CPP1 and CPP2, respectively) (6). The library was prepared using Illumina COVIDSeq assay kit according to guidelines (document no. 1000000126053 v07). Prepared libraries were purified and pooled and then quantified with a Qubit double-stranded DNA (dsDNA) high-sensitivity (HS) assay kit. Libraries were normalized to a 4 nM concentration. Pooled normalized libraries were denatured and finally diluted to a 1.2 nM concentration according to the Illumina Miniseq system denature and dilute libraries guide (document no. 1000000002697 v04). The libraries were then loaded and run on an Illumina MiniSeq instrument following the standard protocol for 150-bp paired-end reads. DRAGEN COVID Lineage app v3.5.8 was used to process and assemble the raw reads as well as for variant calling and lineage determination (7).

The study was approved by the institutional review board (IRB) of Bangabandhu Sheikh Mujib Medical University (IRB no. BSMMU/2021/6137). Written informed consent was taken from the patient before data collection. All the information obtained from the study participants was kept confidential.

In total, 39 SARS-CoV-2 genome sequences were obtained. Sequence identifier (ID), GISAID accession no., GenBank accession no., SRA accession no., total genome length, Nextstrain clade, and Pango Lineage for each sample were listed in Table 1. Amino acid substitutions of each sequence can be viewed online using the following link: https://figshare.com/articles/dataset/Table_2_docx/19613880/1. Genome length across the samples was 29,749 to 29,798 bp. Median coverage ranged from 832× to 2,025×. All sequences were assigned to WHO-designated variant of concern (VOC) Omicron and Nextstrain clades 21K and 21L. Omicron sublineages BA.1, BA.1.1, BA.1.17, and BA.2 were present, among which BA.2 was the most prevalent one.

**TABLE 1:** Sequencing parameters and phylogenetic traits of 39 sequenced genomes

Sequence ID^*a*^	GISAID^*b*^ accession	GenBank accession	SRA^*c*^ accession	Coding-complete length (bp)	Median coverage (×)	GC content (%)	Nextstrain clade	PANGO^*d*^ lineage
BSMMU_Viro-01	EPI_ISL_10894052	ON026021	SRX14879007	29,787	1,588	37.9	21K	BA.1
BSMMU_Viro-02	EPI_ISL_10894053	ON026022	SRX14879008	29,790	953	38	21K	BA.1
BSMMU_Viro-04	EPI_ISL_10894054	ON026023	SRX14879019	29,791	830	38	21K	BA.1.17
BSMMU_Viro-05	EPI_ISL_10894055	ON026024	SRX14879030	29,797	977	38	21K	BA.1
BSMMU_Viro-06	EPI_ISL_10894056	ON026025	SRX14879040	29,771	1,396	37.9	21L	BA.2
BSMMU_Viro-07	EPI_ISL_10894057	ON026026	SRX14879041	29,771	1,236	37.8	21L	BA.2
BSMMU_Viro-10	EPI_ISL_10894058	ON026027	SRX14879042	29,768	1,038	37.9	21L	BA.2
BSMMU_Viro-11	EPI_ISL_10894059	ON026028	SRX14879043	29,768	1,531	37.9	21L	BA.2
BSMMU_Viro-14	EPI_ISL_10894060	ON026029	SRX14879044	29,794	1,870	38	21K	BA.1.1
BSMMU_Viro-16	EPI_ISL_10894061	ON026030	SRX14879045	29,798	1,107	38	21K	BA.1.1
BSMMU_Viro-17	EPI_ISL_10894062	ON026031	SRX14879009	29,758	983	37.9	21L	BA.2
BSMMU_Viro-18	EPI_ISL_10894063	ON026032	SRX14879010	29,771	1,565	37.9	21L	BA.2
BSMMU_Viro-19	EPI_ISL_10894064	ON026033	SRX14879011	29,768	1,528	37.9	21L	BA.2
BSMMU_Viro-20	EPI_ISL_10894065	ON026034	SRX14879012	29,760	1,092	37.9	21L	BA.2
BSMMU_Viro-21	EPI_ISL_10894066	ON026035	SRX14879013	29,776	1,970	37.9	21K	BA.1
BSMMU_Viro-22	EPI_ISL_10894067	ON026036	SRX14879014	29,789	1,561	38	21K	BA.1.1
BSMMU_Viro-23	EPI_ISL_10894068	ON026037	SRX14879015	29,749	832	37.8	21L	BA.2
BSMMU_Viro-26	EPI_ISL_10894069	ON026038	SRX14879016	29,770	1,463	37.9	21L	BA.2
BSMMU_Viro-27	EPI_ISL_10894070	ON026039	SRX14879017	29,771	1,662	37.9	21L	BA.2
BSMMU_Viro-28	EPI_ISL_10894071	ON026040	SRX14879018	29,770	1,302	37.9	21L	BA.2
BSMMU_Viro-30	EPI_ISL_10894072	ON026041	SRX14879020	29,770	1,462	37.9	21L	BA.2
BSMMU_Viro-33	EPI_ISL_10894073	ON026042	SRX14879021	29,786	1,335	38	21K	BA.1
BSMMU_Viro-34	EPI_ISL_10894074	ON026043	SRX14879022	29,789	1,538	37.9	21K	BA.1
BSMMU_Viro-35	EPI_ISL_10894075	ON026044	SRX14879023	29,774	1,521	37.9	21L	BA.2
BSMMU_Viro-37	EPI_ISL_10894076	ON026045	SRX14879024	29,794	1,183	37.9	21K	BA.1.1
BSMMU_Viro-38	EPI_ISL_10894077	ON026046	SRX14879025	29,788	1,508	37.9	21K	BA.1.1
BSMMU_Viro-39	EPI_ISL_10894078	ON026047	SRX14879026	29,754	1,494	37.9	21L	BA.2
BSMMU_Viro-40	EPI_ISL_10894079	ON026048	SRX14879027	29,790	1,229	37.9	21K	BA.1
BSMMU_Viro-41	EPI_ISL_10894080	ON026049	SRX14879028	29,793	1,557	38	21K	BA.1.1
BSMMU_Viro-42	EPI_ISL_10894081	ON026050	SRX14879029	29,791	1,598	37.9	21K	BA.1
BSMMU_Viro-46	EPI_ISL_10894082	ON026051	SRX14879031	29,762	1,442	37.9	21L	BA.2
BSMMU_Viro-47	EPI_ISL_10894083	ON026052	SRX14879032	29,771	1,671	38	21L	BA.2
BSMMU_Viro-49	EPI_ISL_10894084	ON026053	SRX14879033	29,791	1,106	37.9	21K	BA.1
BSMMU_Viro-51	EPI_ISL_10894085	ON026054	SRX14879034	29,763	1,649	37.9	21L	BA.2
BSMMU_Viro-52	EPI_ISL_10894086	ON026055	SRX14879035	29,793	1,766	37.9	21K	BA.1.1
BSMMU_Viro-54	EPI_ISL_10894087	ON026056	SRX14879036	29,749	1,796	37.9	21L	BA.2
BSMMU_Viro-55	EPI_ISL_10894088	ON026057	SRX14879037	29,768	1,651	37.9	21L	BA.2
BSMMU_Viro-56	EPI_ISL_10894089	ON026058	SRX14879038	29,784	1,418	37.9	21K	BA.1.1
BSMMU_Viro-59	EPI_ISL_10894090	ON026059	SRX14879039	29,768	2,025	37.9	21L	BA.2

a ID, identifier.

b GISAID, Global Initiative on Sharing All Influenza Data.

c SRA, Sequence Read Archive.

d PANGO, Phylogenetic Assignment of Named Global Outbreaks.

### Data availability.

The coding-complete genome sequences and metadata of all the 39 samples were submitted to the GISAID database (http://www.gisaid.org) where they can be accessed through the accession no. EPI_ISL_10894052 to EPI_ISL_10894090 (Table 1). The coding-complete genome sequences can be also accessed on the NCBI database (accession no. ON026021 to ON026059, see Table 1). Raw reads were deposited into the SRA database (accession no. were listed in Table 1) under BioProject PRJNA827255.
